# Cholera outbreaks (2012) in three districts of Nepal reveal clonal transmission of multi-drug resistant *Vibrio cholerae* O1

**DOI:** 10.1186/1471-2334-14-392

**Published:** 2014-07-15

**Authors:** Sameer M Dixit, Fatema-Tuz Johura, Sulochana Manandhar, Abdus Sadique, Rajesh M Rajbhandari, Shahnewaj B Mannan, Mahamud-ur Rashid, Saiful Islam, Dibesh Karmacharya, Haruo Watanabe, R Bradley Sack, Alejandro Cravioto, Munirul Alam

**Affiliations:** 1 Center for Molecular Dynamics Nepal, Kathmandu, Nepal; 2 International Centre for Diarrheal Disease Research, GPO Box 128, 1000 Dhaka, Bangladesh; 3 National Institute of Infectious Diseases, Tokyo, Japan; 4 Bloomberg School of Public Health, Johns Hopkins University, Baltimore, MD, USA; 5 International Vaccine Institute, Seoul, Republic of Korea

**Keywords:** Transmission, Antibiotic resistant, Clonal, *V. cholerae*, Cholera, Nepal

## Abstract

**Background:**

Although endemic cholera causes significant morbidity and mortality each year in Nepal, lack of information about the causal bacterium often hinders cholera intervention and prevention. In 2012, diarrheal outbreaks affected three districts of Nepal with confirmed cases of mortality. This study was designed to understand the drug response patterns, source, and transmission of *Vibrio cholerae* associated with 2012 cholera outbreaks in Nepal.

**Methods:**

*V. cholerae* (n = 28) isolated from 2012 diarrhea outbreaks {n = 22; Kathmandu (n = 12), Doti (n = 9), Bajhang (n = 1)}, and surface water (n = 6; Kathmandu) were tested for antimicrobial response. Virulence properties and DNA fingerprinting of the strains were determined by multi-locus genetic screening employing polymerase chain reaction, DNA sequencing, and pulsed-field gel electrophoresis (PFGE).

**Results:**

All *V. cholerae* strains isolated from patients and surface water were confirmed to be toxigenic, belonging to serogroup O1, Ogawa serotype, biotype El Tor, and possessed classical biotype cholera toxin (CTX). Double-mismatch amplification mutation assay (DMAMA)-PCR revealed the *V. cholerae* strains to possess the B-7 allele of *ctx* subunit B. DNA sequencing of *tcpA* revealed a point mutation at amino acid position 64 (N → S) while the *ctxAB* promoter revealed four copies of the tandem heptamer repeat sequence 5*'*-TTTTGAT-3*'*. *V. cholerae* possessed all the ORFs of the Vibrio seventh pandemic island (VSP)-I but lacked the ORFs 498–511 of VSP-II. All strains were multidrug resistant with resistance to trimethoprim-sulfamethoxazole (SXT), nalidixic acid (NA), and streptomycin (S); all carried the SXT genetic element. DNA sequencing and deduced amino acid sequence of *gyrA* and *parC* of the NA^R^ strains (n = 4) revealed point mutations at amino acid positions 83 (S → I), and 85 (S → L), respectively. Similar PFGE (*Not*I) pattern revealed the Nepalese *V. cholerae* to be clonal, and related closely with *V. cholerae* associated with cholera in Bangladesh and Haiti.

**Conclusions:**

In 2012, diarrhea outbreaks in three districts of Nepal were due to transmission of multidrug resistant *V. cholerae* El Tor possessing cholera toxin (*ctx*) B-7 allele, which is clonal and related closely with *V. cholerae* associated with cholera in Bangladesh and Haiti.

## Background

Toxigenic *Vibrio cholerae* is the causative agent of cholera, an acute life-threatening diarrheal disease, which occurs in many developing countries, particularly South Asia, Africa, and Latin America [1,2]. Based on the phenotypic and genotypic differences, *V. cholerae* O1 strains are classified into two biotypes: ‘classical’ and ‘El Tor’ [3]. Seven distinct pandemics of cholera have been recorded since 1817 [4]. The sixth pandemic, and presumably the earlier pandemics, were caused by the classical biotype of *V. cholerae* O1, which was displaced by *V. cholerae* O1 biotype El Tor in the 1960’s to become the causative agent of the ongoing seventh cholera pandemic [1].

Over the past few years, *V. cholerae* O1 biotype ET causing Asiatic cholera has shown remarkable changes in its phenotypic and genetic characteristics [5]. *V. cholerae* O139, also known as Bengal strain emerged in 1992 as the major cause of epidemic cholera in Bangladesh and India by displacing ET biotype strains [6]. *V. cholerae* O1 ET continues to prevail as the major cause of cholera worldwide. The most recent development in the evolution of global cholera has been the emergence and spread of a new variant ET, or altered ET, in Bangladesh that carries *ctxB* of the classical (CL) biotype (*ctxB*^CL^) [7]. Since 2001, *V. cholerae* O1 biotype ET strains associated with endemic cholera in Bangladesh have been altered ET, while those isolated before 2001 contained *ctxB* of the ET biotype [7], which is the prototype associated with the 7th cholera pandemic. According to recent reports, altered ET strains have been spreading globally [8-15] causing more severe disease [16].

Following the earthquake in Haiti in 2010, epidemic cholera broke out in the country that has since affected tens of thousands of people and led to the death of more than 4,000 [17]. Although the 7^th^ cholera pandemic expanded from Asia reaching Africa in the 1970’s and Latin America in 1991, cholera had never been reported in Haiti after 1960 [18]. The source of the recent cholera epidemic in Haiti remains controversial but it is widely believed that it was imported by UN peacekeeping forces arriving from Nepal [17,19].

Nepal is a cholera endemic country where cholera continues to be a major public health concern, especially among lower socio-economic groups. Although cholera causes significant morbidity and mortality each year, Nepal lacks the requisite laboratory infrastructures to conduct epidemiological and related surveillance to determine disease burden, as well as to identify the source and transmission of cholera. Both of these types of information are important for the effective management of all infectious diseases, not only cholera and diarrhea. In 2012, outbreaks of cholera were reported in three districts of Nepal with death of some of the affected individuals in remote villages in the north-western district of Doti. Cholera treatment is often hindered due to emergence of multi-drug resistance in *V. cholerae*[20,21]. Compounding the problem related to selecting an effective antibiotic for cholera treatments, even less is known about drug resistance properties of *V. cholerae* O1 bacterium in Nepal [22-25]. In the present study, *V. cholerae* strains associated with the 2012 cholera outbreaks in three district of Nepal were investigated for microbiological and molecular characteristics. The major tests applied to this study included antibiotic response patterns, and multi-locus genetic screening by polymerase chain reaction, DNA fingerprinting analyses by sequencing, and pulsed-field gel electrophoresis (PFGE) of genomic DNA.

## Methods

### Ethical approval

Ethical approval for the present study was obtained from the Ethical Review Board of Nepal Health Research Council (NHRC) (Reg. 68/2011; 2068-12-20 approved on 2 April 2012). Informed consent was obtained for all patients enrolled in the present study, for patients under 18 years of age parental consent was obtained.

### Isolation of *V. cholerae*

**Figure 1:**
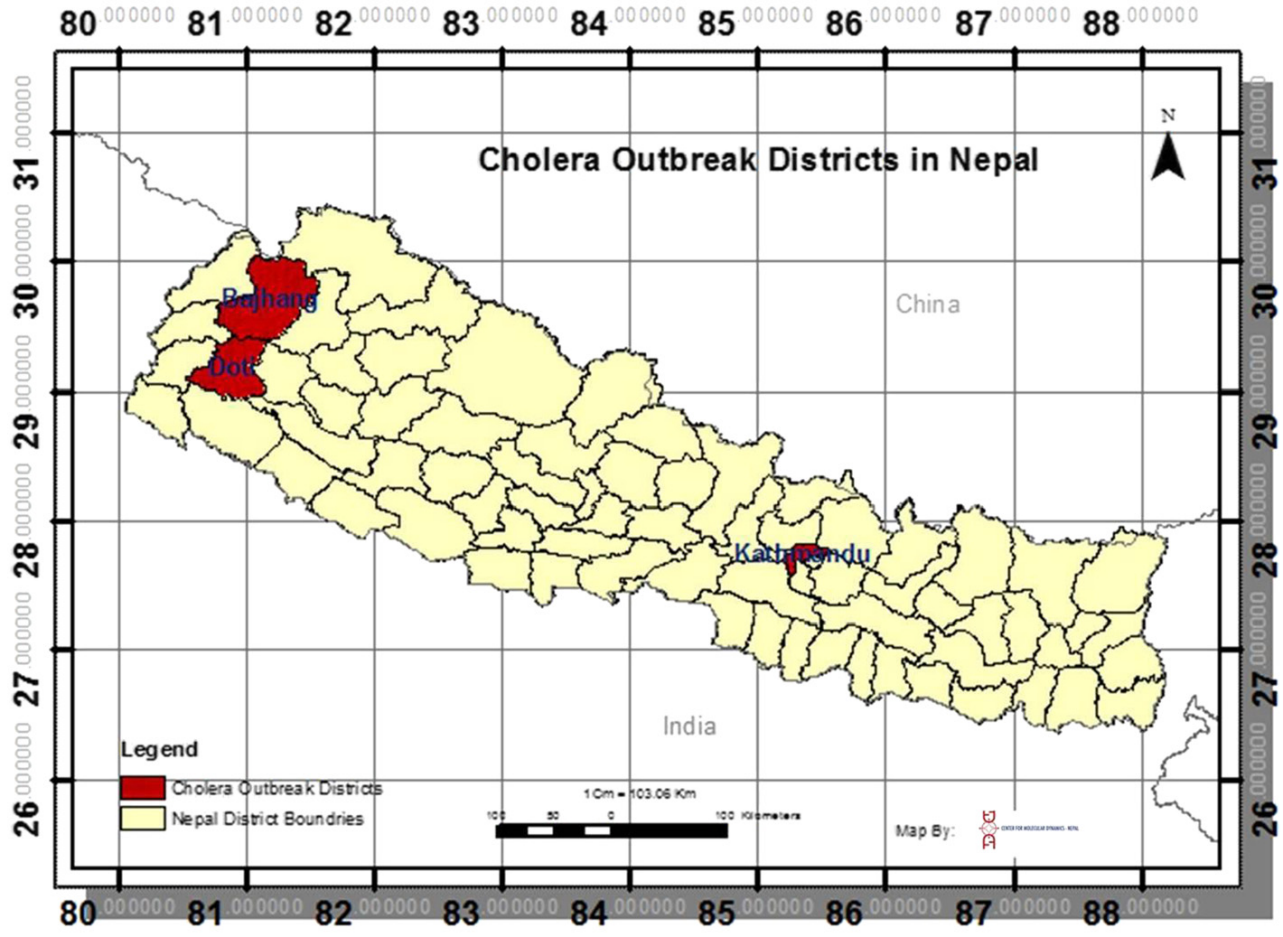
**Map of Nepal showing the cholera affected three districts: Doti, Bajhang, and Kathmandu, 2012.** Cholera affected two north-western districts, Doti and Bajhang, including the capital city, Kathmandu, appear in red.

During the 2012 cholera outbreaks in three districts of Nepal (Figure 1), *V. cholerae* isolated from clinical [Kathmandu (n = 12), Doti (n = 9), and Bajhang (n = 1)] and natural surface water [Kathmandu (n = 6)] sources were confirmed by microbiological, serological and molecular methods. *V. cholerae* isolation involved enriching the samples in alkaline peptone water (APW) (pH-8.4) at 37°C for 4–6 h, followed by culturing overnight on selective bacteriological media such as taurocholate tellurite gelatin agar (TTGA). *V. cholerae* colonies were confirmed using a combination of biochemical and serological methods, as described previously [26]. *V. cholerae* isolates were examined for phenotypic test such as antimicrobial response, as well as for virulence, molecular, and phylogenetic characteristics. Reference *V. cholerae* O1 strains N16961 and O395 served as biotypes El Tor (ET) and Classical (CL) control, respectively.

### Determination of serogroup and biotype

Serogroups of the *V. cholerae* isolates were identified by biochemical and molecular methods and confirmed by slide agglutination using specific polyvalent antisera for *V. cholerae* O1 and O139, followed by screening with a monoclonal antibody specific for both serogroups [26]. Biotyping primarily involved selective phenotypic tests, including chicken erythrocyte agglutination, sensitivity to polymyxin B, and Mukherjee CL phage IV and Mukherjee ET phage V [1].

### Genomic DNA preparation

Genomic DNA extraction was carried out following previously described methods [11].

### PCR assays for serogroup and biotype determination

Subtypes of all strains were reconfirmed using *V. cholerae* species-specific *omp*W PCR [27]. Serogroups were reconfirmed using multiplex PCR targeted at O1- (*rfb*O1) and O139- (*rfb*O139) specific O biosynthetic genes and the cholera toxin (CTX) gene (*ctx*A) [28]. Biotype specific characteristics were determined using PCR assays targeted to *tcpA* (CL and ET) [29], *rstR*, a gene encoding phage transcriptional regulator [30], presence of the repeat in toxin (*rtx*C) [31], *rst*C that encodes an anti-repressor protein, and *tlc*, that codes for the toxin-linked cryptic plasmid [32].

### Determination of *ctxB* genotype

The double mismatch amplification mutation assay (DMAMA-PCR) was recently developed to discriminate the classical (*ctxB* genotype 1), El Tor (*ctxB* gneotype 3), and Haitian types (*ctxB* genotype 7) of *ctxB* alleles by focusing on nucleotide positions 58 and 203 of the *ctxB* gene [33]. DMAMA-PCR was performed in this study to detect the *ctxB* genotype using primers and conditions as described previously [33]. *V. cholerae* O1 strains O395 (CL), N16961 (ET), and 2010EL-1786 (Haiti variant, genotype 7) were used as control strains for DMAMA-PCR analysis.

### Nucleotide sequencing and analysis of genes *ctxB* and *tcpA*

Genes *ctxB* and *tcpA* of randomly selected *V. cholerae* O1 strains in 2012 were sequenced following conditions as described elsewhere [34]. PCR amplification of genes *ctxB* and *tcpA* was performed in a 25 μl reaction mixture in an automated Peltier thermal cycler (PTC-200, M. J. Research). Subsequently, PCR products were purified with a Microcon centrifugal filter device (Millipore Corporation, Bedford, MA) and sequenced using an ABI PRISM Big Dye Terminator Cycle Sequencing Reaction kit (Applied Biosystems, Foster City, CA) on an ABI PRISM 310 automated sequencer (Applied Biosystems). The deduced amino acid sequences of the respective genes from all strains were aligned using CLUSTAL-W.

### The promoter sequence of *ctxAB*^*clas*s^ operon

The 600 bp DNA fragments containing the entire P_*ctxAB*_ of *V. cholerae* strains (n = 4) from Nepal were PCR-amplified using zot gene (the gene preceding the ctx AB operon)-specific forward and ctxA gene-specific reverse primers, ZtPF and CtPR respectively [35], followed by direct sequencing of the amplified products.

### ORF content analysis of VSP-I and VSP-II region

The presence or absence of ORFs in the VSP-I and VSP-II cluster of *V. cholerae* O1 isolates was examined by PCR using primers described elsewhere [32]. *V. cholerae* O1 strains N16961 El Tor (ET) and O395 Classical (CL) biotypes served as controls.

### Antibiotic susceptibility

Susceptibility to antibiotics was performed by disk diffusion, as described by both Bauer *et al*. [36] and the Clinical and Laboratory Standards Institute [37], using commercial antibiotic discs. Nine antibiotics (Oxoid, United Kingdom) were employed: erythromycin (E, 15 μg); gentamicin (CN, 10 μg); trimethoprim/sulfamethoxazole (SXT, 30 μg), tetracycline (TE, 30 μg), ampicillin (AMP, 30 μg), streptomycin (S, 10 μg), azithromycin (AZM, 15 μg), nalidixic acid (NA, 30 μg) and ciprofloxacin (CIP, 5 μg). Characterizations of the resistance or susceptibility profiles of the isolates were determined by measuring the inhibitory zone and comparing it with an interpretative chart to determine sensitivity to each antibiotic.

### PCR assay for the detection of SXT element

Using PCR assays, all *V. cholerae* O1 strains were examined for the presence of the SXT element. The detection of SXT was performed using primers and procedures described previously [38].

### Sequence analysis of *gyrA* and *parC* genes

The DNA sequencing of the PCR amplified genes encoding DNA gyrase (*gyrA*) and topoisomerase IV (*parC*) was performed according to the previously described procedure [39].

### Pulsed-field gel electrophoresis (PFGE)

Whole agarose-embedded genomic DNA from the *V. cholerae isolates* was prepared. PFGE was carried out using a contour-clamped homogeneous electrical field (CHEF-DRII) apparatus (Bio-Rad), according to procedures described previously [40]. Conditions for separation were as follows: 2 to 10s for 13 h, followed by 20 to 25 s for 6 h. An electrical field of 6 V/cm was applied at an included field angle of 120°. Genomic DNA of the test strains was digested by *Not*I restriction enzyme (Gibco-BRL, Gaithersburg, MD), and *Salmonella enterica* serovar Braenderup was digested using *Xba*I, with fragments employed as molecular size markers. Restriction fragments were separated in 1% pulsed-field-certified agarose in 0.5X TBE (Tris-borate-EDTA) buffer. Post-electrophoresis gel-treatment included gel-stained and de-stained. The DNA was visualized using a UV transilluminator, and images were digitized via a one-dimensional gel documentation system (Bio-Rad).

### Image analysis

The fingerprint pattern in the gel was analyzed using a computer software package, Bionumeric (Applied Maths, Belgium). After background subtraction and gel normalization, the fingerprint patterns were typed according to banding similarity and dissimilarity, using the Dice similarity coefficient and unweighted-pair group method employing average linkage (UPGMA) clustering, as recommended by the manufacturer. The results were graphically represented as dendrograms.

## Results

### Phenotypic and genetic characteristics

*V. cholerae* O1 strains (n = 28) included in this study from 2012 diarrhea outbreaks and environmental sources in Nepal produced translucent colonies with black center on TTGA, and gave biochemical reactions typical of *V. cholera*e. All strains reacted positively to serogroup O1-specific antibody, but not to O139, confirming all to be *V. cholerae* O1. Serological results also showed that all *V. cholerae* O1 belonged to the Ogawa serotype (Table 1). All *V. cholerae* strains exhibited chicken cell agglutination (CCA), sensitivity to ET-specific phage V, and resistance to both polymyxin B and CL specific phage IV, confirming biotype El Tor (ET)-specific phenotypic traits (Table 1).

**Table 1:** **Phenotypic, genotypic and drug resistance properties of *V*. *cholerae *** **O1 isolated in Nepal (n = 28), 2012**

**Country**	**District**	**Year of isolation**	**No. of isolates**	**Source**	**Serotype**	***wbe*** **O1**	**Phenotypic properties**				**Genetic screening by PCR**							**Resistance profile**	***sxt***
							**Sensitivity**												
							**CCA**	**PMB (50U)**	**CL -specific phage IV**	**El Tor -specific phage v**	***ctxA***	***tcpA type***	***ctxB***^***a ***^ ***genotype***	***rstR type***	***rtxC***	***rstC***	***tlc***		
Nepal	Doti	2012	9	Clin	Ogawa	+	+	R	R	S	+	ET	B7	ET	+	+	+	SXT, NA, S	+
	Kathmandu	2012	6	Env	Ogawa	+	+	R	R	S	+	ET	B7	ET	+	+	+	SXT, NA, S	+
	Kathmandu	2012	12	Clin	Ogawa	+	+	R	R	S	+	ET	B7	ET	+	+	+	SXT, NA, S	+
	Bajhang	2012	1	Clin	Ogawa	+	+	R	R	S	+	ET	B7	ET	+	+	+	SXT, NA, S	+
India		1965	O395	Clin	Ogawa	+	-	S	S	R	+	CL	B1	CL	-	-	+		
Bangladesh		1971	N16961	Clin	Inaba	+	+	R	R	S	+	ET	B3	ET	+	+	+		

^a^Determined by double mismatch amplification mutation assay (DMAMA) PCR (Naha *et al*., [33]); Clin, clinical; Env, environmental; PMB, polymyxin B; R, resistant; s, sensitive; ET, El Tor; CL, classical; CCA, chicken cell agglutination SXT, trimethoprim/sulfamethoxazole; NA, Nalidixic Acid; S, Streptomycin.

The phenotypically confirmed *V. cholerae* O1 biotype ET strains amplified the primers for species-specific gene *ompW*, and all amplified primers specific for O biosynthetic gene *wbe* of *V. cholerae* O1, but not *wbf*, which is specific for serogroup O139 (Table 1). All of the *V. cholerae* O1 strains isolated from cholera and from surface water sources in Nepal amplified the primers for the cholera-toxin gene *ctxA* (Table 1). In addition, all of the *ctxA*^+^*V. cholerae* strains amplified the primers for biotype ET-specific marker gene *rtxC*, confirming ET attributes. All strains had *tcpA*, the major virulence-associated gene of the VPI-I gene cluster, and all amplified primers for ET-specific marker gene *tcpA*^ET^, but not *tcpA*^CL^. All of the *V. cholerae* ET strains confirmed in this study carried the ET biotype-specific repressor gene *rstR*^ET^, confirming that they carried ET-biotype CTX prophage, and possessed *rstC* and *tlc* genes (Table 1).

### *CtxB* typing by DMAMA-PCR

All *V. cholerae* O1 strains isolated from both clinical and environmental sources (n = 28), including the O395 (CL), N16961 (ET) and 2010EL-1786 (Haiti variant, *ctxB* genotype 7) were analyzed using double-mismatch amplification mutation assay (DMAMA)-PCR technique to determine the CTX-B genotype. As shown in Table 1, all of the ET Biotype strains amplified the primers specific for *ctxB* genotype 7 irrespective of their source and place of isolation.

### Sequencing of *ctxB* and *tcpA*

PCR-amplified genes *ctxB* (460 bp) and *tcpA* (675 bp) from selected *V. cholerae* O1 strains (n = 4; one clinical strain from each of the three districts including one environmental strain from Kathmandu) were sequenced and the N’-amino acid sequences determined using bioinformatic tools. Sequencing data revealed that all of the tested *V. cholerae* O1 strains contained the deduced amino acid sequence of CTXB. This is identical to that of the CL biotype CT, having histidine and threonine at positions 39 and 68, respectively, but an additional sequence variation was observed in position 20, where histidine found in CL and ET biotype CT was replaced by asparagine (H → N) (Nucleotide accession numbers are KJ596538, KJ596539, KJ596540 and KJ596541). The DNA sequence and deduced amino acids matched against the *ctxB* genotype 7. The *ctxB* sequencing data were consistent and supported the results of DMAMA-PCR.

DNA sequencing data of the amplified *tcpA* gene revealed the presence of a point mutation that resulted in an amino acid substitution at position 64 (N → S) of the deduced peptide (accession numbers are KJ596546, KJ596547, KJ596548 and KJ596549).

### The promoter sequence of *ctxAB*^*clas*s^ operon

Analysis of the promoter sequences of the randomly selected Nepalese *V. cholerae* O1 strains isolated in 2012 revealed that they contained four copies of heptamer repeat sequences in the P_*ctxAB*_ regions (accession numbers are KJ596554, KJ596555, KJ596556 and KJ596557) irrespective of their source of isolation. This indicates that the CT regulatory regions of these strains are like those of canonical El Tor strains and dissimilar to those of classical strains, which usually carry seven to eight such repeats [38].

### VSP Islands

All Nepalese *V. cholerae* O1 examined in this study carried all the tested ORFs of the VSP-I genomic island region. However, all strains lacked the VSP-II genomic island ORFs VCO498, VCO502, VCO504 and VCO511 (Table 2).

**Table 2:** **ORF content analysis of VSP islands of *V*. *cholerae *** **O1 isolated in Nepal (n = 28), 2012**

				**VSPI**					**VSPII**											
**Year of isolation**	**No. of isolates**	**District**	**Source**	**VCO175**	**VCO178**	**VCO180**	**VCO183**	**VCO185**	**VCO490**	**VCO493**	**VCO498**	**VCO502**	**VCO504**	**VCO511**	**VCO512**	**VCO513**	**VCO514**	**VCO515**	**VCO516**	**VCO517**
2012	9	Doti	Clin	+	+	+	+	+	+	ND	-	-	-	-	ND	+	+	+	ND	+
2012	6	Kathmandu	Env	+	+	+	+	+	+	ND	-	-	-	-	ND	+	+	+	ND	+
2012	12	Kathmandu	Clin	+	+	+	+	+	+	ND	-	-	-	-	ND	+	+	+	ND	+
2012	1	Bajhang	Clin	+	+	+	+	+	+	ND	-	-	-	-	ND	+	+	+	ND	+
1965	O395		Clin	-	-	-	-	-	-	-	-	-	-	-	-	-	-	-	-	+
1971	N16961		Clin	+	+	+	+	+	+	+	+	+	+	+	+	+	+	+	+	+

Clin, clinical; Env, environmental; ND, not done.

### Antibiotic susceptibility assay

Antibiotic susceptibility assay revealed that all of the 28 tested *V. cholerae* O1 strains isolated in 2012 from the three districts of Nepal, irrespective of their source and place of isolation, were multi-drug resistant with resistance to NA, SXT, and S. However, all strains were sensitive to AZM, TE, AMP, CN, E, and CIP (Table 1).

### Detection of SXT element

All of the *V. cholerae* strains (n = 28) amplified the primer for the SXT gene, a mobile genetic element carrying multi-drug resistance gene cassettes in bacteria (Table 1).

### Sequencing of *gyrA* and *parC*

Sequencing of *gyrA* and *parC* of *V. cholerae* O1 strains (n = 4) representing three districts of Nepal, including both clinical and environmental sources of Kathmandu, detected one point mutation in each; in *gyrA* serine was substituted by isoleucine at position 83 (accession numbers are KJ596542, KJ596543, KJ596544 and KJ596545), and in *parC* serine was substituted by leucine at position 85 (accession numbers are KJ596550, KJ596551, KJ596552 and KJ596553).

### PFGE and cluster analysis

**Figure 2:**
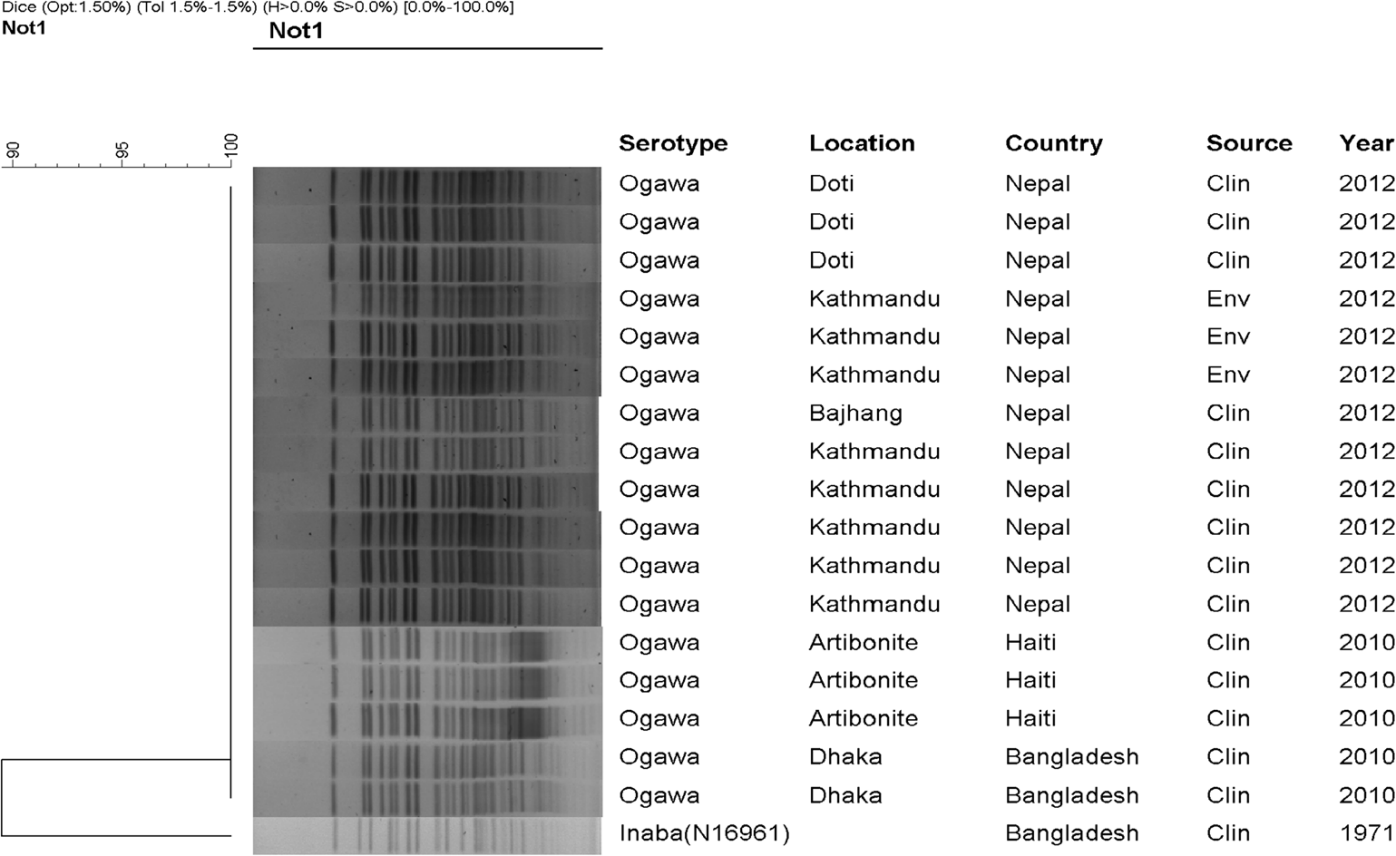
**Dendrogram showing genomic fingerprints of *V*. *cholerae *O1 strains isolated in Nepal in 2012.** Genomic fingerprinting of multi-drug resistant *V. cholerae* O1 El Tor strains isolated from 2012 diarrhea outbreaks in three districts (Doti, Bajhang, and Katmandu) of Nepal. Dendrogram was constructed by Dice similarity coefficient and UPGMA clustering method by using pulsed-field gel electrophoresis (PFGE) images of *NotI*- restriction digested genomic DNA of the tested Nepalese *V. cholerae* strains; PFGE images of contemporary *V. cholerae* O1 El Tor strains from Haiti and Bangladesh, including El Tor reference strain N16961 were also included. The scale bar at the top (left) indicates similarity coefficient. The banding patterns and the similarity coefficient revealed 100% similarity for all the tested Nepalese, Haitian, and Bangladeshi *V. cholerae* strains included in the dendrogram, suggesting° high level of clonal relatedness (Data available from the Dryad Digital Repository: doi:10.5061/dryad.600nd).

The PFGE of *Not*I-digested genomic DNAs of the *V. cholerae* O1 strains yielded 20 to 23 fragments (Figure 2) (Data available from the Dryad Digital Repository: doi:10.5061/dryad.600nd), and their molecular sizes ranged from 20.5 to 350 kb. The PFGE pattern of all the Nepalese strains irrespective of their source and place of isolation matched both with each other, and with that of the altered El Tor PFGE pattern in the number and position of the DNA fragments, suggesting genetic homogeneity .

In order to understand the clonal link between the *V. cholerae* O1 strains associated with cholera outbreaks in Doti, Kathmandu, and Bajhang, cluster analysis was performed by dendogram using the PFGE (*Not*I) images of the Nepalese *V. cholerae* O1 strains, together with PFGE (*Not*I) images of representative *V. cholerae* O1 strains isolated in Bangladesh (2010) and Haiti (2010), which were available in our soft database. All of the Nepalese strains were clonal as they shared the same cluster showing 100% similarity, and were related closely with representative *V. cholerae* O1 altered El Tor strains isolated in Bangladesh and Haiti.

## Discussion

This study presents microbiological and molecular data on *V. cholerae* isolated from 2012 Nepal cholera and surface water sources showing the transmission of a highly clonal, multi-drug resistant *V. cholerae* O1, which was associated with simultaneous cholera outbreaks occurring in Kathmandu and the remote villages of the western districts of Doti and Bajhang, Nepal. The study also revealed very close clonal relationships between the Nepalese *V. cholerae* O1 strains with those from Bangladesh and Haiti isolated in 2010.

The conventional culture, biochemical, and serological test results confirmed that both clinical and environmental *V. cholerae* isolates belonged to serogroup O1 and serotype Ogawa. The microbiological test results were complemented by the molecular data obtained from PCR assays performed for the amplification of *V. cholerae* species-specific gene *ompW*[27], genes *ctxA* encoding subunit A of cholera toxin (CTX), and *wbe* encoding serogroup O1-specific antigenic polysaccharides [28]. These results confirmed that all of the *V. cholerae* isolates were toxigenic and belonged to serogroup O1. The phenotypic characteristics, together with the presence of biotype specific marker genes, such as *rtxC*, *rstC*, *tcpA*^ET^, *rstR*^ET^, and *ctxB*^CL^, confirmed that all of the *V. cholerae* O1 strains occurring in surface water and associated with the 2012 cholera outbreaks in Nepal were biotype El Tor possessing the *ctxB* marker gene of classical biotype. This important data identified all strains as altered El Tor, first described in Bangladesh in 2006 [7,11].

Although treatment of cholera involves a course of effective antibiotics, together with appropriate oral or intravenous rehydration fluid(s) [41], antibiotic therapy world-wide has faced with challenges related to the rapid emergence and spread of multi-drug resistant (MDR) *V. cholerae* strains resistant to atibiotics, such as TE, AMP, kanamycin (KN), S, SXT, NA, E, and most recently, to CIP and norfloxacin (NOR) [17,21,42,43]. In Nepal, at least three different resistance phenotypes of *V. cholerae* were previously reported to be in circulation [24]. A recent study reported temporal variation in drug resistance patterns of *V. cholerae* associated with cholera in Nepal between 2007 and 2010 [25]. In the present study, *V. cholerae* O1 associated with diarrheal outbreaks in Nepal, including those isolated from natural surface water samples, were MDR showing resistance towards SXT, NA, and S, suggesting that drug resistance patterns can change temporally, and spatially, and thus require continuous monitoring in order to select an effective drug of choice at any given time.

In *V. cholerae*, multi-drug resistance was shown to be attributed to lateral acquisition of self-transmissible genetic element designated SXT, carrying multiple antibiotic-resistance markers [44]. All *V. cholerae* strains tested in the present Nepalese study were MDR and likewise, all had the SXT element, presumably carrying MDR marker genes in their genome, a fact that appears to be in line with their consistent resistance towards trimethoprim-sulfamethxazole. Resistance to fluoroquinolones such as NA is mostly associated with genes encoding gyrase (*gyrA* and gyrB) and topoisomerase IV (*parC* and *parE*). DNA sequencing of the *gyrA* and *parC* of the Nepalese *V. cholerae* strains suggests a similar molecular basis for quinolone resistance, as a single point mutation in each of the genes resulted in amino acid switching from Serine to Isoleucine at 83 position, and Serine to Leucine at 85 position, respectively, as found in the quinolone resistant *V. cholerae* associated with cholera in Africa [20], India [39], and Haiti [45].

The polymorphism of *ctxB* gene-encoding cholera toxin (CT) subunit B and the corresponding amino acid substitution was first reported in the early 1990’s [34]. Subsequent investigation of the *ctxB* gene sequence revealed the presence of eleven distinct genotypes in different serogroups of *V. cholerae*[46]. Genotypes 1, 2, 3, 7, 10, and 11 were found in serogroup O1 strains, genotypes 3, 4, 5, and 6 were found in serogroup O139 strains, and genotypes 8 and 9 were found only in serogroups O27 and O37 [46]. A previous study showed the presence of two different *ctxB* genotypes, namely 1 and 7, among *V. cholerae* associated with cholera in Nepal between 2007 and 2010 [25]. In the present study, *ctxB* genotype 7 was confirmed for all 28 *V. cholerae* O1 strains isolated in 2012 from three districts of Nepal. Data presented in this study provide ample evidence of genetic switching from *ctxB* genotype 1 to 7 in Nepal, a phenomenon seen in 2007 and 2008 for *V. cholerae* causing endemic cholera in India [47] and Bangladesh [48], respectively. The global dissemination of *ctxB* genotype 7 has been growing with reports of the genotype in the West African countries of Nigeria and Cameroon, as well as in Haiti [17,20].

The toxin co-regulated pilus (TCP), an essential colonization factor of *V. cholerae*[49] that also serves as a receptor for CTX-Φ [44], is a homopolymer of the major pilus protein, TcpA pilin [49] encoded by *tcpA*. The DNA sequence of *tcpA* differs slightly at the C-terminal domain for classical and El Tor biotype strains [3]. In the present study, DNA sequencing and the deduced amino acid sequences of TcpA of *V. cholerae* O1 El Tor strains associated with cholera outbreaks in Nepal (2012) differed from the El Tor reference strain N16961 due to a mutation at the amino acid position 64 (Asparagine → Serine). This change may be subtle and it is not known whether such genetic switching of the *tcpA* gene has any epidemiological impact on *V. cholerae* causing endemic cholera in Nepal. Nonetheless, a change of amino acid at position 64 of TcpA was first reported in the *V. cholerae* serogroup O1 biotype El Tor strain associated with cholera in Bangladesh [50]. This was followed by reports from Haiti [51], suggesting that this genotype is spreading globally.

ToxR is a global transcriptional regulator protein responsible for virulence gene expression, and *toxR* sequence repeats (TTTTGAT) located between CTX prophage genes *zot* and *ctxA* are essential for ToxR binding and activation of the *ctxAB* promoter [52]. The Nepalese *V. cholerae* O1 strains contained four copies of such repeats (data not shown), while five copies were found among the El Tor variant of *V. cholerae* associated with cholera in Haiti [17,45]. These are less than the seven copies that were reported for the classical biotype strains of *V. cholerae* O395.

The VSP-I gene cluster encompasses a 16 kb region from VC0175 to VC0185, and most of the genes encode hypothetical or conserved proteins with no known function. On the other hand, the VSP-II region is a ~27 kb region that encompasses VC0490–VC0516 [29]. These two clusters are unique to the El Tor strains of the seventh pandemic. The Nepalese *V. cholerae* O1strains carried intact VSP-I but harbored a variant VSP-II lacking the ORFs 498–511 of the VSP-II genomic island region, which was first reported from a *V. cholerae* strain (CIRS101) associated with cholera in Bangladesh [53] and Haiti [45].

A recent MLVA-based study carried out with *V. cholerae* O1 strains associated with cholera between 2007 and 2010 showed the circulation of four different groups of altered *V. cholerae* O1 El Tor strains in Western Nepal including Butwal and Kathmandu [25]. The molecular basis and epidemiological significance of such genetically divergent *V. cholerae* O1 altered El Tor remains an interesting area to explore. However, *V. cholerae* O1 associated with cholera outbreaks in the three Nepalese districts proved to be highly clonal, since all of the strains had an indistinguishable PFGE (*Not*-I) banding pattern irrespective of their source of isolation, reflecting high genetic homogeneity in the *V. cholerae* population. The PFGE pattern generated by the *V. cholerae* O1 strains in this study also matched with the pattern reported for *V. cholerae* O1 associated with cholera outbreaks in Bangladesh and Haiti, suggesting that closely related multi-drug resistant strains are undergoing global dissemination.

## Conclusion

In conclusion, this study presents data on the transmission of a multi-drug resistant *V. cholerae* showing identical PFGE pattern in three districts (Doti, Bajhang, and Kathmandu) of Nepal during the 2012 diarrhea outbreaks. Considering the changing climate and increasing global burden of cholera, and the emergence and spread of new hyper-infective variants of *V. cholerae*[16], regular surveillance of *V. cholerae* outbreaks is highly recommended for monitoring the distribution, clonal type and evolution of the pathogen, for effective cholera management in Nepal and elsewhere.

## Competing interests

The authors declare that they have no competing interests.

## Authors’ contributions

SMD, MA, AC, designed and HW and RBS supported the study. MA coordinated and SMD, FTJ, SM, AS, SBM, MUR, and SI carried out the study. SMD, RMR, and DK arranged the sample collection. SMD, FTJ, and MA analyzed data and prepared the manuscript. AC, HW, and RBS helped in manuscript writing and revision. All authors reviewed and approved the final version of the manuscript.

## Pre-publication history

The pre-publication history for this paper can be accessed here:

http://www.biomedcentral.com/1471-2334/14/392/prepub
